# Unfavorable Effects of Peritoneal Dialysis Solutions on the Peritoneal Membrane: The Role of Oxidative Stress

**DOI:** 10.3390/biom10050768

**Published:** 2020-05-14

**Authors:** Stefanos Roumeliotis, Evangelia Dounousi, Marios Salmas, Theodoros Eleftheriadis, Vassilios Liakopoulos

**Affiliations:** 1 Division of Nephrology and Hypertension, 1st Department of Internal Medicine, School of Medicine, Aristotle University of Thessaloniki, 54636 Thessaloniki, Greece; st_roumeliotis@hotmail.com; 2 Department of Nephrology, Faculty of Medicine, School of Health Sciences, University of Ioannina, 45110 Ioannina, Greece; evangeldou@gmail.com; 3 Department of Anatomy, School of Medicine, National and Kapodistrian University of Athens, 11527 Athens, Greece; salmasmarios5@gmail.com; 4 Department of Nephrology, University Hospital of Larissa, 41110 Larissa, Greece; teleftheriadis@yahoo.com

**Keywords:** glucose degradation products, oxidative stress, peritoneal dialysis, peritoneal membrane, reactive oxygen species

## Abstract

One of the main limitations to successful long-term use of peritoneal dialysis (PD) as a renal replacement therapy is the harmful effects of PD solutions to the structure and function of the peritoneal membrane (PM). In PD, the PM serves as a semipermeable membrane that, due to exposure to PD solutions, undergoes structural alterations, including peritoneal fibrosis, vasculopathy, and neoangiogenesis. In recent decades, oxidative stress (OS) has emerged as a novel risk factor for mortality and cardiovascular disease in PD patients. Moreover, it has become evident that OS plays a pivotal role in the pathogenesis and development of the chronic, progressive injury of the PM. In this review, we aimed to present several aspects of OS in PD patients, including the pathophysiologic effects on the PM, clinical implications, and possible therapeutic antioxidant strategies that might protect the integrity of PM during PD therapy.

## 1. Introduction

Peritoneal dialysis (PD) is a cost-effective, high-quality renal replacement therapy (RRT) used by approximately 196,000 end-stage renal disease (ESRD) patients worldwide, representing 11–15% of the dialysis population [1]. Although PD remains a life-sustaining therapy with increasing utilization in countries, such as China and the USA, during the past decade, the proportion of ESRD patients treated with PD declined in many parts of Europe and other developed countries [2]. This wide disparity in the utilization of PD might be partially attributed to financial and commercial reasons that favor hemodialysis (HD) or by a lack of patient knowledge about dialysis modalities. Limited understanding about PD therapy or a fear of complications and side-effects could represent other major drawbacks for the expansion of PD programs. One of the main limitations to successful long-term use of the method is the deleterious effects of PD solutions to the structure and function of the peritoneal membrane (PM), leading to loss of dialysis capacity. The peritoneum is the largest biological internal membrane, covering the surface of abdominal organs and the walls of the abdominal cavity. It is composed of a monolayer of mesothelial cells, supported by connective tissue, underneath of which there are blood vessels, nerves, and lymphatic vessels. In PD, the PM serves as a semipermeable membrane that might present structural alterations due to exposure to PD solutions, including peritoneal fibrosis, vasculopathy, and neoangiogenesis. Chronic exposure eventually leads to a progressive degradation of the PM in approximately 50 to 80% of all PD patients, which is accompanied by inability to remove excessive water, salt, and uremic toxins. Inadequate dialysis triggers malnutrition, infection, and cardiovascular (CV) morbidity and mortality, whereas insufficient water removal might result in fluid retention, hypertension, and heart failure [3].

Therefore, it is essential to fully elucidate the exact pathophysiologic mechanisms underlying the deleterious alterations of the PM during long-term PD, in order to recognize therapeutic targets to protect the membrane integrity. In this direction, oxidative stress (OS) has emerged during the past two decades as a novel non-traditional, uremia-related risk factor for increased mortality and morbidity in PD patients. Moreover, in these patients, there is accumulating evidence suggesting a pivotal role of OS in the pathogenesis of the chronic PM damage. In this review, we aimed to present several aspects of OS in PD patients, including the pathophysiologic effects on the PM, clinical complications, and possible therapeutic antioxidant strategies that might protect the integrity of PM during PD therapy.

## 2. OS in PD: The Composition of PD Solutions Is the Main Culprit

OS is defined as the imbalance between pro-oxidants and antioxidants in favor of the former, targeting proteins, lipids, carbohydrates, and nucleic acids and, subsequently, causing organ injury and dysfunction [4]. Among pro-oxidant molecules, reactive oxygen species (ROS) are the most common, whereas antioxidants (such as vitamins C, E, and *N*-acetylcysteine-NAC) are molecules with the ability to neutralize free pro-oxidants and can be either endogenously generated or exogenously supplemented. OS is present at early stages of Chronic Kidney Disease (CKD) and progresses parallelly to the deterioration of kidney function; compared to healthy subjects, patients at CKD stage 1–4 have an excess of OS status, whereas compared to stage 4, stage 5 ESRD patients present even higher OS status [5]. RRTs further exacerbate OS, which is mainly attributed to procedure-related factors [6]. Compared to PD, HD patients present significantly higher production of ROS and increased depletion of antioxidants, mainly due to blood exposure to bioincompatible dialyzer and dialysate. Other factors promoting OS in HD include use of heparin, intravenous iron infusion, use of central venous catheters, and dysfunctional arteriovenous fistulae, as well as loss of antioxidant vitamins and trace elements during HD sessions [7]. Although OS has been thoroughly studied in HD, the data regarding the pathophysiologic mechanisms, diagnosis, and management of OS in PD are quite limited. However, there is evidence suggesting that OS in PD patients is increased more than in pre-dialysis ESRD patients. This increase occurs despite the absence of contact of blood with artificial materials, use of heparin, or vitamin loss during dialysis and limited use of intravenous iron, key oxidative stress enhancers in HD. Therefore, OS in PD patients is probably triggered by different pathophysiologic mechanisms than HD. The main source of OS in PD is considered to be the non-physiologic composition of conventional PD solutions, including high glucose concentration, increased osmolarity, and acidic pH [8]. The composition of PD solutions is toxic for the peritoneal mesothelial cells (PMCs) and causes both acute and chronic morphologic and functional damage in the PM. In vitro human PMCs cannot survive more than 40 min when cultured with conventional PD solutions, whereas the prolonged exposure in vivo to the hostile, bioincompatible PD fluids causes fibrosis, vasculopathy, and loss of peritoneal ultrafiltration [9]. The main factor causing acute and chronic peritoneal damage is the non-physiologic, hyperglycemic content of PD fluids.

## 3. High-Glucose, Glucose Degradation Products, and Advanced Glycation End-Products

Hyperglycemia is the main determinant for development of diabetic microvascular complications. It has been hypothesized that hyperglycemia results in peritoneal damage through pathophysiologic mechanisms, similar to those that lead to glomerular diabetic injury. During the heat sterilization of PD solutions, glucose degradation products (GDPs) are accumulated in the dialysate. When the PMCs are exposed to the bioincompatible PD fluid characterized by high glucose and GDPs concentrations, GDPs undergo further non-enzymatic chemical reactions and become irreversibly heterogenous reactive molecules, the advanced glycation end-products (AGEs). GDPs affect the PM either directly or indirectly through the increased accumulation of AGEs in the PM. As PD fluid enters the peritoneal cavity, AGEs upregulate the expression of their specific multi-ligand, transmembrane receptor (RAGE), and accumulate around the PM. Intracellular formation of AGEs cause peritoneal cell injury through three mechanisms. AGEs cause a morphologic modification of intracellular proteins, modify the structure of extracellular matrix components and receptors expressed on the surface of the peritoneal cells, and, finally, AGEs and AGE-modified proteins bind tightly to RAGE on macrophages and endothelial cells. This binding triggers formation of ROS, which in turn activate pro-inflammatory molecules, cytokines, transcriptional, and growth factors, such as nuclear factor kappa-light-chain-enhancer of activated B cells (NF-kB) and vascular endothelial growth factor (VEGF), leading to abnormal transcription of DNA and apoptosis. Moreover, the free radicals that are generated by the AGEs-RAGE interaction further upregulate the generation of AGEs, forming a vicious cycle. The OS state in the peritoneal cavity is a combined result of overproduction of ROS and impaired antioxidant defense mechanisms. The extent of the glycoxidation-derived peritoneal cell damage depends on other parameters, as well, such as genetic factors that may pre-determine the characteristics of the PM and the extent of natural antioxidant defense mechanisms and the presence of systematic disorders, like hypertension, diabetes, and dyslipidemia [10].

The main pathway through which GDPs and AGEs trigger formation of ROS is the increase glucose-oxidation metabolism. The increased glucose influx into the peritoneal cells cause an increase in glucose catabolism through the Krebs cycle and a subsequent overproduction of electron donors at quantities that overwhelm the capacity of endogenous mechanisms to neutralize them [11]. Subsequently, electrons are donated (one at a time) to molecular oxygen, formatting ROS. In vitro studies showed that exposure of human PMCs to conventional PD solutions (containing 1.5, 2.5, and 4.25% dextrose) for 1 h was accompanied by a significant formation of ROS, disruption of the mitochondrial membrane and PMC death [12]. Several other mechanisms of hyperglycemia-induced production of ROS in peritoneal cells have been proposed, including enhanced metabolism of arachidonic acid, increased expression of polyol pathway, and increased production of diacylglycerol and activation of protein kinase C of PMCs [13]. In turn, free radicals formed during mitochondrial metabolism, cause oxidative damage in proteins, carbonyls, lipids, and DNA. Oxidative injury of mitochondrial DNA accumulates due to the impaired repair mechanism of this damaged DNA. All types of cells lining or living near the peritoneal cavity (including PMCs, endothelial, and leukocytes), after long-term exposure to the toxic environment of high glucose, GDPs, AGEs, ROS, and inflammatory mediators undergo gradual and irreversible morphological and functional changes that lead to their death. However, PMCs, under chronic exposure to toxic PD fluids might either detach from the PM or undergo a morphological transition to mesenchymal phenotype that includes abolishment of their polarized cytoskeletal structure and transformation to a myofibroblast phenotype with increased motility [14,15,16]. These transformed cells have the ability to secrete extracellular matrix compound, angiogenic and fibrotic factors [17]. Moreover, various types of cell death have been reported in peritoneal cells exposed to PD solutions, including anoikis, apoptosis, primary and secondary necrosis, and mitotic catastrophe [18]. Apoptotic death of PMCs mainly derives from oxidative DNA and protein injury. To explore the pathophysiologic mechanisms linked to cell death in PD patients, Simon et al. obtained human PMCs from an overnight lavage of 7 stable patients undergoing PD with high-glucose hyperosmotic PD solutions and found that PMC death was solely triggered by the formation of intracellular OS [19]. Similarly, the main factor triggering death of peritoneal leukocytes and endothelial cells is the enhanced oxidative genomic damage. Compared to the other types of cells, endothelial cells are more sensitive to OS and display a higher rate of apoptotic death [20]. In vitro incubation of human PMCs in conventional 4.25% dextrose PD solutions is accompanied by shrinkage of the PMCs within the first minute of incubation. Over 60% of the cells suffer apoptotic death within 2 h, due to the deleterious effects of GDPs and OS [21,22]. Similarly, in another in vitro study, GDP-derived oxidation of DNA caused apoptotic death of PMCs in a time and dose-dependent manner [23]. The pivotal role of DNA oxidative damage in the long-term complications of PD was also highlighted by Ishibashi et al. The authors obtained peritoneal tissue samples from 10 long-term, stable, continuous ambulatory PD (CAPD) patients, 5 controls with normal renal function, and 3 ESRD, pre-dialysis patients and incubated them in conventional PD solutions with 56 mmol/L and 222 mmol/L glucose. Histopathology of PM from CAPD patients exhibited a strong cytoplasmic staining for 8-hydroxy-2′-deoxyguanosine (8-OH-dG), a critical biomarker assessing the effect of endogenous oxidative DNA, whereas ESRD patients presented only faint staining and no staining was found in subjects with normal renal function. Moreover, only PMCs of CAPD patients exhibited strong staining for 8-OH-dG, whereas PMCs of ESRD patients and subjects with normal renal function showed only faint staining. The staining was more pronounced in the high glucose solution. Immunoelectron microscopy found that 8-OH-dG was localized in mitochondrial DNA [24]. Similar results were reported in another in vitro study, where human PMCs were incubated for 24 h with 5 mM glucose (control) and 236 mM (equal to the glucose concentration of a hypertonic conventional 4.25% PD solution), with and without supplementation of the antioxidant NAC. Compared to the control group, exposure to high glucose concentration resulted in a 46.3-fold increase in hydrogen peroxide (a well-known free radical), a 4.9-fold increase in malondialdehyde (MDA), a marker of lipid peroxidation, and a 21.4-fold increase in mitochondrial 8-OH-dG levels. The hyperglycemic-induced oxidation of oxygen, lipids and DNA was accompanied by mitochondrial fragmentation. Moreover, NAC (a well-known antioxidant) successfully decreased the production of ROS and MDA, preserved the integrity of PM, rescued mitochondria from fragmentation and abrogated PMCs’ apoptosis in a dose-dependent manner [23]. In agreement with these results, incubation of peritoneal endothelial cells in a hyperglycemic environment also caused enhanced generation of ROS and OS-induced apoptosis [25]. Similarly, intraperitoneal administration of hypertonic dextrose PD solution in rats for 4 weeks, caused overproduction of MDA and VEGF and decreased concentrations and activity of the antioxidant glutathione peroxidase. This accelerated OS status caused structural alterations of the PM, including neoangiogenesis and increased thickness, which in turn was accompanied by skewed peritoneal function tests. The authors concluded that the enhanced hyperglycemia-induced OS in the peritoneal cavity is a result of upregulation of pro-oxidants and downregulation of antioxidants and is the main determinant of PM degradation [26]. The detrimental effects of high glucose PD solutions were also reported in peritoneal white blood cells; GDPs at concentrations found in conventional hypertonic 4.25% dextrose PD solutions promoted caspase-dependent apoptosis of peritoneal white blood cells [27].

**Figure 1:**
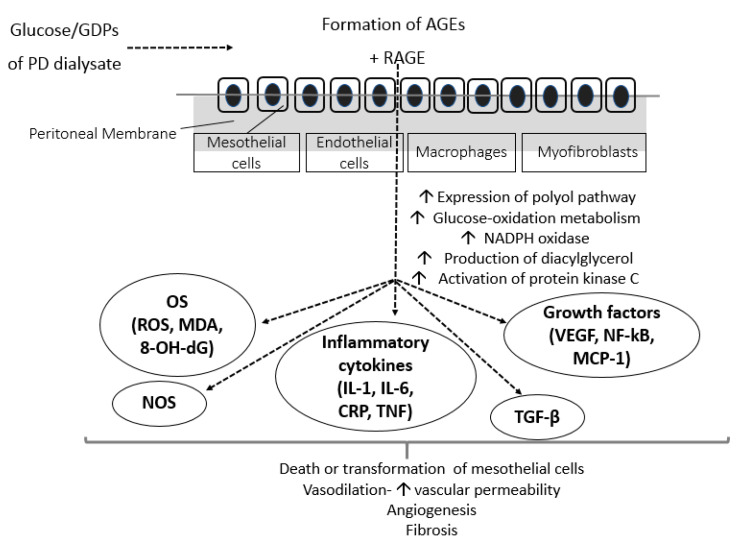
Effects of conventional hyperglycemic PD solutions on the peritoneum. AGEs, advanced glycation end-products; CRP, c-reactive protein; GDPs, glucose degradation products; IL, interleukin; MCP-1, monocyte chemoattractant protein-1; MDA, malondialdehyde; NADPH, nicotinamide adenine dinucleotide phosphate; NF-kB, nuclear factor kB; NOS, nitric oxide synthase; PD, peritoneal dialysis; RAGE, AGE-receptor; TGF-β, transforming growth factor β; TNF, tumor necrosis factor; VEGF, vascular endothelial growth factor; 8-OH-dG, 8-hydroxy-2′-deoxyguanosine.

There is a growing body of evidence from experimental, animal and human studies showing that the free radicals formed due to prolonged exposure of all types of peritoneal cells to the high-glucose PD solutions, further stimulate activation of growth and transcriptional factors, such as NF-kB, VEGF, monocyte chemotactic peptide-1 (MCP-1), and tumor growth factor-β (TGF-β) [13,28]. In turn, activation of these factors leads to accelerated accumulation of extracellular matrix, leading to PM fibrosis and, subsequently, to PM degradation and loss of ultrafiltration. Moreover, PD-derived vasculopathy upregulates the complement system and its regulatory pathways. The level of arteriolopathy and the degree of exposure to hyperglycemic PD fluids were strongly associated with the activation of complement in peritoneal specimens of children on long-term PD [29]. MCP-1 is a chemokine responsible for monocyte migration to PM, production of extracellular matrix, and formation of collagen by fibroblasts that has been associated with systemic inflammation, all-cause, and CV mortality when measured in dialysate of PD patients [30]. Among all cytokines involved, TGF-β appears to be the most crucial mediator of hyperglycemic OS-derived expansion of extracellular matrix. In vivo studies have shown that, compared to 5.6 mmol/L glucose (control), both animal and human PMCs cultured with high glucose (50 mmol/L) exhibited a 1.7- to 3.1-fold increase in expression of TGF-β and fibronectin mRNA and a 1.5-fold increase in MCP-1 mRNA expression. Stimulation of these growth and fibrotic factors was induced by hyperglycemic oxidative alterations in PMCs, depending on the concentrations of glucose in a dose-dependent manner [31,32]. Recent data also suggest that miR-21 (one of the most abundant miRNA found in PMCs) is a crucial effector of PM fibrosis and could be a novel biomarker of PM transformation in the PD effluent of patients undergoing PD [33]. In vitro studies in human PMCs showed that hyperglycemic-induced upregulation of TGF-β suppressed the expression of Matrix Gla Protein, a well-established inhibitor of ectopic vascular calcification [34,35]. Combet et al. obtained peritoneal biopsies from 7 healthy controls and 13 patients undergoing PD either for a short (<18 months) or long (>18 months) period and found that, compared to controls, long-term PD patients exhibited a 5-fold increase in endothelial nitric oxide synthase activity (attributed to accumulation of AGEs) and increased endothelial liberation of VEGF. The biologic relevance of endothelial upregulation of VEGF in long-term PD was demonstrated by a significant increase in the endothelial area and enhancement of angiogenesis, vasodilation, and vascular permeability of the peritoneum, all of which cause deterioration of the PM [36]. Moreover, hyperglycemia-induced generation of ROS also triggers local and systemic chronic inflammation status that contribute to the deleterious effects of OS in the PM [37]. It has been shown that within the first 15 min of PD solution entry in the peritoneum, production of inflammatory markers, such as C-reactive protein (CRP) and interleukin-6 (IL-6), is triggered [38]. In turn, inflammatory molecules promote production of free radicals and both local and systemic arteriosclerosis. The effects of high glucose and GDPs in the peritoneum are depicted in Figure 1.

## 4. The Effect of Acidic pH and Lactate Buffer on Peritoneal OS

Although GDPs and AGEs play pivotal role, they are not the exclusive source of enhanced OS and inflammation in the peritoneal cavity. It has been shown in vitro that acidic PD solutions reduced the viability of PMCs and peritoneal leukocytes [39]. In a study in PD patients, a low pH PD solution triggered an immediate (within the first minute) release of iron from transferrin. In the acidic pH solution, free iron caused an enhanced oxidative response of the red blood cells’ membrane, assessed by an abrupt increase in thiobarbituric acid reactive substances (TBARS) generation (a marker of lipid peroxidation status) and protein carbonylation. Moreover, accumulation of iron was prominent in areas of the peritoneum with increased fibrosis. On the contrary, in neutral pH PD solution, release of iron was not observed. The authors concluded that neutralizing the PD solution might help avoid the enhanced OS-damage triggered by iron release from transferrin [40]. Similarly, in vitro exposure of human PMCs to 7.5% icodextrin and standard acidic pH PD solutions containing 1.5, 2.5, and 4.25% dextrose resulted in excessive formation of ROS and cell death. However, adjustment of the acidity, significantly attenuated the PD-derived OS and PMC apoptosis, however, only in icodextrin and 1.5% dextrose solutions [12]. In agreement with these findings, adjustment of PD solution pH from acidic-5.2 to neutral-7.3 state reversed the detrimental effects caused by the acidic environment to human PMCs [38]. However, the fibrotic changes in the PM of rats treated with neutral or acidic pH PD solutions did not differ significantly. The authors concluded that acidic pH causes OS, independently of glucose by-products, but less fibrosis of the PM.

Lactate buffer is considered another bioincompatible factor in PD solutions. In vitro cell metabolism of human PMCs incubated with PD solution, have been shown to be significantly improved with bicarbonate compared to lactate buffer. At a standard pH of 7.4, a bicarbonate-containing medium is superior to a lactate-containing medium for preserving mitochondrial activity, PM integrity and PMC viability and preventing expression of fibrotic factors by these cells [41]. These results were attributed to the fact that compared to bicarbonate, lactate buffer is more bioincompatible for the peritoneum and promotes significantly increased generation of AGEs, ROS and upregulation of inflammatory mediators [42]. Moreover, it was shown that the in vitro expression and function of human peritoneal mesothelial aquaporin-1 was regulated by buffer agents and pH used in PD fluids. Incubation of human PMCs with bicarbonate-buffered PD fluids upregulated aquaporin-1 expression, which in turn led to enhanced PMC migration capacity, whereas incubation with lactate PD fluids reduced the expression of aquaporin-1 [43]. In animal models treated with PD solutions for 8–12 weeks, compared to lactate-buffered, bicarbonate/lactate buffered PD solution resulted in significantly less degree of submesothelial fibrosis [28], decreased angiogenesis, reduced OS status and superior performance of the PM [44]. It has also been reported that the improved biocompatibility of bicarbonate/lactate buffered (compared to standard lactate buffered) PD solutions is independent of pH [44]. To investigate the effect of PD fluid buffer on the function of PM, Schmitt et al. conducted a multicenter, randomized, controlled trial in 37 children on PD. All patients were randomly allocated to neutral-pH, low-GDP PD solutions with either 35 mM lactate or 34 mM bicarbonate as buffers. After 10 months, the authors found that the acidosis status did not differ among groups. However, compared to lactate, PD biocompatible fluids with bicarbonate content improved long-term preservation of the PM function [45]. Moreover, compared to lactate, bicarbonate-containing PD fluids has been reported to be superior in preservation of peritoneal ultrafiltration in children on chronic PD. This favorable effect of bicarbonate PD solutions was hypothesized to be due to upregulation of angiopoietin-1 and promotion of vessel maturation [46].

## 5. Other PD-Specific Factors Related with OS

The increased OS in PD patients is not solely attributed to the non-physiologic PD solutions. During PD, PM is exposed to plastic, artificial PD catheters and a plethora of uremic toxins, factors triggering formation of free radicals and upregulation of cytokines and inflammatory mediators. However, these factors have not been associated with fibrosis of the PM. Moreover, during long-term PD treatment, several patients experience acute peritoneal infections that also favor generation of pro-oxidants. OS might be also involved in the pathogenesis and clinical course of peritoneal infections. Nitric oxide, a marker of endothelial dysfunction and OS, has been proposed as a marker to assess the severity of PD peritonitis, its course and prognosis [47]. During peritonitis episodes, PM is exposed to a massive infiltration of leukocytes, cytokines, inflammatory and chemotactic molecules and it is possible that a single severe episode of peritonitis could cause irreversible acute injury of all cell types of the PM [48]. In response to appropriate stimuli (including acute infection or chronic exposure to toxic PD fluids), peritoneal polymorphonuclear leukocytes and phagocytes consume molecular oxygen and produce superoxide (a free radical) in a reaction termed respiratory burst. This process is mediated by the nicotinamide adenine dinucleotide phosphate (NADPH-oxidase) dependent molecular pathway. NADPH-oxidase is assembled in the membranes of activated leukocytes and pumps electrons into the cell to catalyze the reduction of oxygen to superoxide anion, which further releases hydrogen peroxide. In turn, these two free radicals have the ability to produce additional ROS, such as singlet oxygen and hydroxyl radical [49]. Moreover, inflammation contributes to the enhanced OS through the NF-kB, tumor necrosis factor-α (TNF-α) and nitric oxide synthase (NOS) pathways [50]. However, it has been shown, in children undergoing PD with neutral-pH, low-GDP PD fluids, that PD vintage and exposure to the toxic hyperglycemic environment and not episodes of peritonitis lead to transformation of PM during the first three years of PD [51]. Preservation of residual renal function (RRF) is of clinical importance in PD patients. GDPs can also affect the function of renal tubular cells, whereas AGEs promote development and progression of diabetic glomerulosclerosis. However, through unknown mechanisms, increased RRF in PD patients has been related with reduced oxidation of proteins and lipids and increased OS status is an independent predictor of RRF deterioration [52]. There is some evidence that abdominal pressure is associated with adverse clinical events and mechanical complications in the course of PD [53,54]. Especially in PD patients that underwent abdominal surgery or those with severe heart failure, abdominal distention might induce hemodynamic changes and ischemia with a subsequent upregulation of growth factors, which in turn might contribute to the chronic degradation of the PM [55,56]. In automated PD, the intraperitoneal pressure per volume of dialysate is lower compared to CAPD [57]. However, to-date, no association has been found between different PD modalities and OS status or degree of PM injury. Compared to CAPD and continuous cyclic PD, nocturnal intermittent PD has been shown to protect against local inflammation, which is intra-related with OS. The favorable anti-inflammatory effect of this modality has been attributed to the increased resting period of the peritoneum during the day [58].

**Figure 2:**
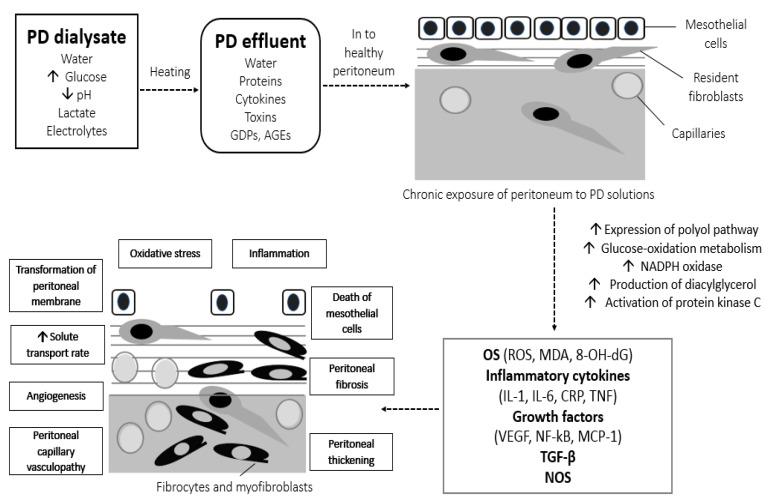
Local and systemic OS-derived complications of conventional hyperglycemic PD solutions. AGEs, advanced glycation end-products; CRP, c-reactive protein; GDPs, glucose degradation products; IL, interleukin; MCP-1, monocyte chemoattractant protein-1; MDA, malondialdehyde; NADPH, nicotinamide adenine dinucleotide phosphate; NF-kB, nuclear factor kB; NOS, nitric oxide synthase; OS, oxidative stress; PD, peritoneal dialysis; ROS, reactive oxygen species; TGF-β, transforming growth factor β; TNF, tumor necrosis factor; VEGF, vascular endothelial growth factor; 8-OH-dG, 8-hydroxy-2′-deoxyguanosine.

Besides the local complications of long-term PD, we can overlook the fact that glucose by-products, free radicals, growth factors, cytokines, and inflammatory mediators that are chronically accumulated in the PM might also result in crucial systemic complications, as shown in Figure 2. Inflammatory and oxidative molecules could be absorbed into the systemic circulation along with glucose by-products, causing systemic inflammation, OS, and various metabolic complications, including increase in body mass index, hyperinsulinemia, insulin resistance, dyslipidemia, development of diabetes, atherosclerosis, and CVD [59]. Moreover, during long-term PD, ROS induced by use of PD solutions might play a role to the pathophysiological alterations in abdominal fat and cause a dysregulation of adiponectin [60]. The morphological and functional alterations of the peritoneum result in loss of peritoneal ultrafiltration rate, fluid and salt overload, insufficient toxin removal, and failure of the PD technique.

## 6. Approaches to Ameliorate OS in PD and Preserve the Integrity of PM

The standard, hyperosmolar, lactate-buffered, acidic-pH, high GDPs-containing PD solutions are responsible for the increased local OS and inflammation seen in PD and the subsequent detrimental effects in PM. Therefore, the improvement of the biocompatibility of PD solutions might represent a promising strategy to suppress OS, preserve the integrity of PM and improve patient outcomes [48].

To investigate the possible cytoprotective advantages of low-GDP PD solutions, Lai et al. performed human PMCs culture experiments in traditional PD and new PD solutions with lower GDP content. Compared to traditional, low GDP PD fluids triggered lower upregulation of growth factors and significantly improved cell viability and OS status in a dose-dependent manner [61]. Mortier et al. divided 36 rats in 3 groups that were injected twice daily, intraperitoneally with different PD solutions for 12 weeks: group 1 received a traditional 3.86% glucose solution, with lactate buffer at pH 5.5, group 2 received a low-GDP, bicarbonate/lactate solution containing 3.86% glucose at pH 7.4, and group 3 received a lactate-buffered amino-acid solution at pH 6.7. The low-GDP rats did not develop submesothelial fibrosis, ultrafiltration loss, activation of growth factors, and formation of AGEs, which were reported in the standard GDP solutions group [28]. Moreover, in human studies, low-GDP PD solutions were associated with improved preservation of PMCs viability and PM integrity and decreased local inflammation and endothelial damage compared to standard solutions [62]. Szeto et al. randomized 50 new PD patients to standard lactate-buffered PD solution (control group) or lactate-buffered, neutral pH, low-GDP solution (active group) for 1 year. After the follow-up period, compared to control, the low-GDP group exhibited significantly increased PD fluid CA-125 (a mesothelial cell marker) and decreased serum CRP (a marker of systemic inflammation, associated with enhanced OS) [63]. The authors concluded that low-GDP PD solutions might preserve the integrity of PM, through suppression of AGEs and ROS formation.

Besides GDPs and AGEs, the acidic pH of PD fluids is an independent determinant of OS-derived damage of the PM. After 2 h incubation of human PMCs in conventional 4.25% dextrose PD solution with acidic pH 5.3, PMCs viability was <40%. The authors modified the pH of the PD solution by adding sodium bicarbonate to a physiologic value of 7.4 and found a significant increase in the survival time of PMCs [22]. To minimize generation of GDPs and maintain a neutral pH, newer, biocompatible PD solutions were developed. Since production of GDPs during heat sterilization of PD fluids is significantly reduced in acidic pH, these new solutions are stored within dual-chambered bags, where glucose is kept in a separate chamber from other electrolytes in acidic conditions (pH 2.8–4.2). Before use, the contents of the chambers are mixed, yielding a final PD solution with neutral pH (6.8–7.3) and containing either low or ultralow GDP content. Conventional PD solutions have an acidic pH (5–5.4) and increased 3,5-dideoxy-glycosone-3-ene (the most bio-reactive GDP) concentration (12–19 μmol/L), whereas low solutions have concentrations of 0.2–11 μmol/L, respectively [64]. However, even low GDP concentrations are still biologically relevant. Data from clinical studies showed an improved biocompatibility of the new PD fluids, assessed by improved markers in PD effluent, such as CA-125; markers of interstitial integrity, such as hyaluronan; markers of neoangiogenesis, such as VEGF; and pro-inflammatory mediators, such as IL-6 [65]. There is a growing body of evidence showing that neutral pH, low GDP, bicarbonate-buffered solutions have important clinical advantages over conventional PD solutions, such as preservation of RRF. The favorable effects of the new PD fluids on peritoneal ultrafiltration, hospitalization technique, and patient survival are still under debate [66]. The clinical benefits of neutral pH, low GDP PD fluids over conventional, acidic pH, high GDP solutions were also highlighted in a recent meta-analysis of 29 studies and 1971 PD patients. [67]. The novel PD fluids resulted in better preservation of RRF, but their effects on hard end-points, such as hospitalization, occurrence of peritonitis, and adverse events remain uncertain. The Euro-Balance Trial was a multicenter, open-label, prospective randomized controlled trial with a crossover design aiming to compare the effects of standard, acidic pH, high GDP (control) and neutral pH, low-GDP PD solutions (balance group) on the PM. Eighty-six PD patients were randomized to either the control or the balance group for 12 weeks and then switched to opposite groups for a period of another 12 weeks. The authors found that the use of neutral pH, low-GDP fluids resulted in significantly reduced levels of AGEs, as well as improvement in effluent biomarkers of PM integrity. Moreover, the balance group presented an improvement in RRF, with an accompanying reduction in peritoneal ultrafiltration [68]. In agreement with these results, 8 years later, Johnson et al. performed the balANZ trial a multicenter, open-label, prospective randomized controlled trial that aimed to investigate the effect of neutral pH-low GDP PD solutions (Balance group) compared to standard GDP solutions (control group) on PM function. One-hundred eighty five incident PD patients were allocated to either the Balance group (85 patients) or the control group (82 patients) and followed for 2 years. Use of the Balance solutions was associated with an initially increased PM transport rate and reduced peritoneal ultrafiltration, compared to the control group. During the follow-up period, peritoneal ultrafiltration rate increased significantly and all other parameters remained stable in the Balance group, whereas there was a progressive deterioration in PM permeability in the control group, over time [69]. Moreover, this study reported that the new PD fluids exerted a protective effect against infectious complications and were more cost-effective than the conventional fluids [70]. The development of more biocompatible, low-GDP, neutral pH PD solutions is a significant improvement. New, randomized controlled trials evaluating the different effects of conventional and novel PD fluids on PD-induced alterations in the function of PM and clinical hard end-points are needed. However, the International Pediatric Peritoneal Biopsy Study Group obtained biopsy samples from 110 children undergoing long-term PD (85% on low-GDP solutions) and found detrimental alterations in the structure of PM (including inflammation, fibrosis, and vasculopathy) with low-GDP solutions, quite similar to those previously reported with conventional high GDP solutions in adults [65]. Similarly, in a large cohort study including 90 ESRD children at the time point of PD catheter insertion and 82 children undergoing PD, it was revealed that neutral pH-low GDP PD solutions induce crucial, early changes of the PM, development of fibrosis, and transformation of PMC to mesenchymal phenotype [71]. Therefore, it has become evident that there is still a great medical need to improve the biocompatibility of PD fluids [65].

To counteract the favorable effect of high osmolarity of PD solutions on OS, icodextrin, an iso-osmolar PD solution with decreased concentration of GDPs was developed. The use of icodextrin (a glucose polymer) has important clinical benefits over conventional glucose PD solutions, including increased ultrafiltration, decreased incidence of fluid overload, and improved lipid profile. Icodextrin may probably protect the structure and function of PM, compared to conventional glucose PD solutions. Compared to conventional, 1.36% dextrose PD solution, in vitro use of glucose-free PD fluids (containing icodextrin or amino-acids) was accompanied by a significant less formation of AGEs and reduced production of various markers of carbonyl oxidation [72]. In disagreement with these findings, Gotloib et al. found that 30 days exposure of rat PMCs to icodextrin resulted in accelerated lipid-peroxidation status that caused disruption of PM and oxidative DNA injury [73]. To determine whether icodextrin is accompanied by improvement of OS status and less cytotoxicity in vivo, mice were injected intraperitoneal with 7.5% icodextrin (once daily for one month). Through upregulating lipid and carbonyl oxidation, icodextrin induced a marked genomic oxidative injury, leading to death of PMCs [74]. Similarly, the use of icodextrin triggered enhanced local inflammation status and oxidative damage leading to DNA damage and, subsequently, to death of all types of PM cells in CAPD patients [20,75,76]. Therefore, the biocompatibility of icodextrin cannot be considered much superior to that of a conventional glucose PD solution, in regard to peritoneal inflammation, OS and cell death. Newer, more biocompatible, iso-osmolar PD solutions are needed in order to suppress OS and preserve the integrity of all peritoneal cells.

Since the culprit for the accelerated OS seen in PD is the overproduction of ROS and the reduced activity of antioxidants, several investigators hypothesized that local supplementation of antioxidants could ameliorate the OS in these patients [77] and therefore might preserve the integrity of the PM and even exert clinical benefits [78]. NAC is a thiol-containing powerful ROS scavenger with favorable results when administered per OS in dialysis patients [79]. In vitro administration of NAC in human PMCs, with exhibited hyperglycemic-induced OS and apoptosis, successfully decreased the oxidative damage of lipids and DNA [23]. Similarly, administration of NAC significantly attenuated the hyperglycemic-induced formation of ROS and abrogated their negative metabolic effects [60]. Moreover, NAC and the antioxidant vitamin E have been shown to disrupt the hyperglycemic-induced activation of NF-kB by ROS and suppress production of free radicals and therefore ameliorate both OS and fibrogenesis in PMCs cultured with high glucose PD solutions [13]. Du et al. incubated human endothelial cells in hyperglycemic solutions and found an increased formation of ROS, upregulation of NF-kB, and induction of apoptosis that started 2 h after the incubation. All these effects of a hyperglycemic environment were prevented by administration of the antioxidant tocopherol [25]. Administration of pyruvate, a scavenger of the free radical hydrogen peroxide, to rat PMCs that were incubated in glucose solution resulted in significant suppression of OS [80]. In vivo long-term PD rats were divided to receive treatment with conventional high-glucose, lactate-buffered PD solution or pyruvate-buffered solution for 1 month. Compared to conventional, pyruvate solution was accompanied by significantly less degree of neoangiogenesis, inflammation, OS, and fibrosis of the PM. Under the effects of pyruvate, PMCs demonstrated normal density, structure, and function and were almost similar to PMCs before exposure to PD fluids [81]. The favorable, antioxidative effects of pyruvate were also present in human PMCs incubated at both neutral and acidic pH and at normal or high glucose conditions [82]. To investigate the possible beneficial effects of trimetazidine on the glucose-induced alterations of the PM, Gunal et al. divided rats in those that were treated with a conventional hypertonic, high dextrose solution and those that received the same solution but with intraperitoneal coadministration of trimetazidine. After 4 weeks, the first group exhibited hyperglycemic-derived structural and functional alterations in PM, attributed to enhanced local OS. However, compared to the first group, the trimetazidine group exhibited significantly lower degree of PM thickness and neoangiogenesis, accompanied by reduced levels of MDA and VEGF and upregulated activity of the antioxidant glutathione peroxidase [26]. There are numerous other agents proposed to protect the integrity of PM through amelioration of OS, apoptosis, inflammation, and fibrosis, including: carnitine and alanyl-glutamine (AlaGln) dipeptide, low molecular weight heparin, sodium sulfite, hyaluronan, aminoguanides, phosphatidylcholine, l-2-oxothiazolidine, and prothiols [83]. However, the evidence regarding the effects of these agents is scarce and only derived from small experimental studies. Among other agents, there is a growing body of evidence from cell cultures and clinical studies suggesting that addition of AlaGln in the PD solution might exert a beneficiary protective effect on peritoneal mesothelium, through several cytoprotective mechanisms, including reduction of local OS status. In vitro exposure of PMCs to conventional PD solutions aggravated cytotoxic injury and suppressed cytoprotective cellular stress responses (CSR) [84]. Moreover, in vitro data showed that supplementation of PD fluids with AlaGln restored the cytoprotective stress proteome and enhanced the activity of PMCs’ defense mechanisms against the toxic PD fluids-induced injury [85]. Since AlaGln was already used in critically ill patients for parenteral nutrition, the same group of investigators performed the first human study to determine the possible beneficiary effects of AlaGln supplementation on CSR in PD patients. In this randomized cross-over clinical trial, addition of 8 mM of AlaGln in a conventional, acidic PD solution successfully restored peritoneal glutamine levels, host defense, and CSR of PMCs, without affecting small solute transport, peritoneal ultrafiltration or pro-inflammatory biomarkers [86]. Moreover, supplementation of AlaGln in the PD fluids resulted in decreased activity of PM-damage mechanisms and restoration of cytoprotective defense mechanisms [87]. In a double-blind, randomized, placebo-controlled, cross-over trial, addition of 8 mM AlaGln for 2 months into a low-GDP, neutral-pH PD solution, significantly improved biomarkers of inflammation and PM integrity [88]. It has also been shown that, targeted metabolomic profiling of PD effluents showed that AlaGln addition to PD fluids was accompanied by a significant decrease of methionine sulfoxide, a biomarker of OS [89]. A recent study investigated the effects of both conventional and neutral pH, PD solutions with and without addition of AlaGln on peritoneal surface composition, and proteome during chronic PD, in a well-established animal model. Chronic exposure of the peritoneum of rats to PD solutions activated pathways linked to cell differentiation and injury, abnormal metabolism, immune responses, inflammation, and OS. However, AlaGln addition to PD fluids successfully deactivated the pathways associated with PM degradation and activated pathways associated with protection of the PM integrity [90].

## 7. Conclusions

Preservation of the PM integrity in long-term PD, is crucial for maintaining survival of both patients and the dialysis technique. Compared to other patients with ESRD, including pre-dialysis patients, PD patients exhibit accelerated OS, due to factors different from those seen in HD patients. In PD, the bioincompatible composition of PD fluids (including high glucose, high osmolarity, and acidic pH) is the main factor for promoting OS. GDPs and AGEs contained in conventional PD solutions trigger formation of ROS through several molecular pathways. In turn, ROS promote accumulation of pro-inflammatory markers, growth and fibrotic factors in the PM. Exposure of the PM in this highly toxic environment leads to detrimental effects on the structure and function of PS, with severe local and systemic complications. To suppress hyperglycemic-induced OS and preserve the integrity of PM, a multitargeted strategy is warranted. In this direction, the development of new, more biocompatible, iso-osmolar, neutral pH, low-GDP, bicarbonate-buffered PD solutions is needed. Moreover, in vitro and clinical data support that addition of AlaGln in the PD fluids might attenuate the detrimental effects of chronic PD on the PM. Future, well-designed randomized controlled studies will establish the beneficiary effects of these solutions on clinical hard endpoints.

## Abbreviations/Nomenclature

**Abbreviation**	**Definition**
8-OH-dG	8-hydroxy-2’-deoxyguanosine
AGEs	Advanced glycation end-products
AlaGln	Alanyl-glutamine
CAPD	Continuous ambulatory peritoneal dialysis
CKD	Chronic kidney disease
CRP	C-reactive protein
CSR	Cytoprotective cellular stress responses
CV	Cardiovascular
ESRD	End-stage renal disease
GDPs	Glucose degradation products
HD	Hemodialysis
IL-6	Interleukin-6
MCP-1	Monocyte chemotactic peptide-1
MDA	Malondialdehyde
NAC	*N*-acetylcysteine
NADPH	Nicotinamide adenine dinucleotide phosphate
NF-kB	Nuclear factor kappa-light-chain-enhancer of activated B cells
NOS	Nitric oxide synthase
OS	Oxidative stress
PD	Peritoneal dialysis
PM	Peritoneal membrane
PMC	Peritoneal mesothelial cell
RAGE	Receptor for AGEs
ROS	Reactive oxygen species
RRF	Residual renal function
RRT	Renal replacement therapy
TBARS	Thiobarbituric acid reactive substances
TGF-β	Tumor growth factor-β
TNF-α	Tumor necrosis factor-α
VEGF	Vascular endothelial growth factor
